# Supplementary material for: Investigation of refractive index dynamics during in vitro embryo development using off-axis digital holographic microscopy
Source: Biomed Opt Express. 2023 Jun 13;14(7):3327–42. doi: 10.1364/BOE.492292 (PMC10368053; doi:10.1364/BOE.492292)
Supplement: Supplementary file 1 [file boe-14-7-3327-s001.pdf]

Supplemental Document

Biomedical Optics EXPRESS

Investigation of refractive index dynamics during *in vitro* embryo development using off-axis digital holographic microscopy: supplement

GEORGE O. DWAPANYIN,[1,†] DARREN J. X. CHOW,[2,3,4,†] TIFFANY C. Y. TAN,[2,3,4] NICOLAS S. DUBOST,[1] JOSEPHINE M. MORIZET,[1] KYLIE R. DUNNING,[2,3,4,7,‡] AND KISHAN DHOLAKIA[1,5,6,‡,*]

[1]*SUPA, School of Physics and Astronomy, University of St Andrews, North Haugh, St Andrews, Fife, United Kingdom*
[2]*Robinson Research Institute, School of Biomedicine, The University of Adelaide, Adelaide, Australia*
[3]*Australian Research Council Centre of Excellence for Nanoscale Biophotonics, The University of Adelaide, Adelaide, Australia*
[4]*Institute for Photonics and Advanced Sensing, The University of Adelaide, Adelaide, Australia*
[5]*School of Biological Sciences, The University of Adelaide, Adelaide, Australia*
[6]*Centre of Light for Life, The University of Adelaide, Adelaide, Australia*
[7]*kylie.dunning@adelaide.edu.au*
[†]*These authors contributed equally.*
[‡]*These authors contributed equally.*
[*]*kishan.dholakia@adelaide.edu.au*

This supplement published with Optica Publishing Group on 13 June 2023 by The Authors under the terms of the Creative Commons Attribution 4.0 License in the format provided by the authors and unedited. Further distribution of this work must maintain attribution to the author(s) and the published article's title, journal citation, and DOI.

Supplement DOI: https://doi.org/10.6084/m9.figshare.23266313

Parent Article DOI: https://doi.org/10.1364/BOE.492292

1. FIGURES AND TABLES

Table S1. **Sample sizes for the different embryo developmental stages imaged**

	Low lipid	High lipid
2-Cells	11	14
4-Cells	22	21
8-Cells	22	37
Morula	29	42
Blastocysts	28	15

Table S2. **Average measured refractive indices for each developmental stage**

	2-cell	4-cell	8-cell	Morula	Blastocysts
Low lipid	1.347	1.346	1.347	1.346	1.344
High lipid	1.349	1.348	1.349	1.351	1.346

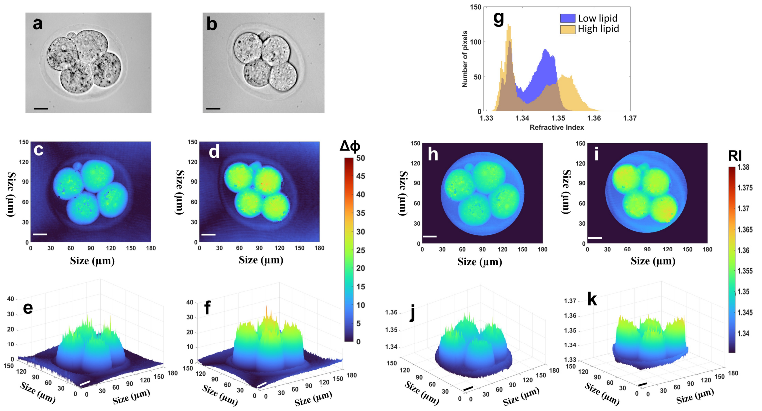

Fig. S1. Representative phase information and refractive index maps for 4-cell embryos cultured in low or high lipid media. (a,b) Bright field images for low lipid and high lipid-treated 4-cell embryos, respectively. (c,e and d,f) Reconstructed 2D phase profiles and their corresponding 3D topographical profiles for low lipid and high-lipid group respectively. (h,j and i,k) Reconstructed 2D refractive index profiles for low lipid and high lipid group with their corresponding topographical refractive index profiles. (g) Frequency distribution of all pixels measured using the reconstructed 2D refractive index profiles for low and high lipid groups of embryos in (h and i, respectively). Scale bars = 30 μm.

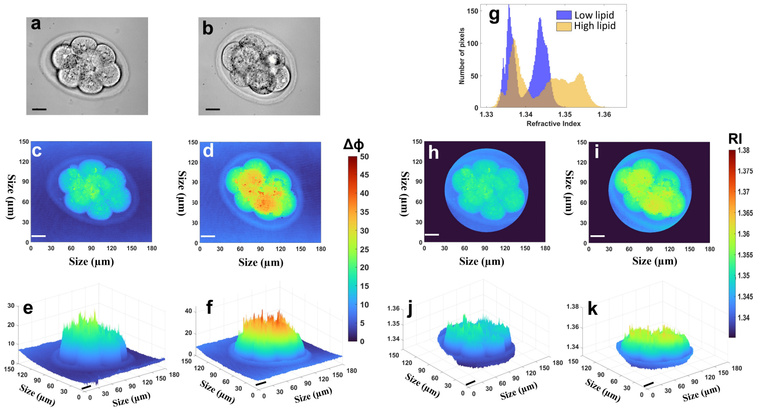

Fig. S2. Representative phase information and refractive index maps for 8-cell embryos cultured in low or high lipid media. (a,b) Bright field images for low lipid and high lipid-treated 8-cell embryos, respectively. (c,e and d,f) Reconstructed 2D phase profiles and their corresponding 3D topographical profiles for low lipid and high-lipid group respectively. (h,j and i,k) Reconstructed 2D refractive index profiles for low lipid and high lipid group with their corresponding topographical refractive index profiles. (g) Frequency distribution of all pixels measured using the reconstructed 2D refractive index profiles for low and high lipid groups of embryos in (h and i, respectively). Scale bars = 30 μm.

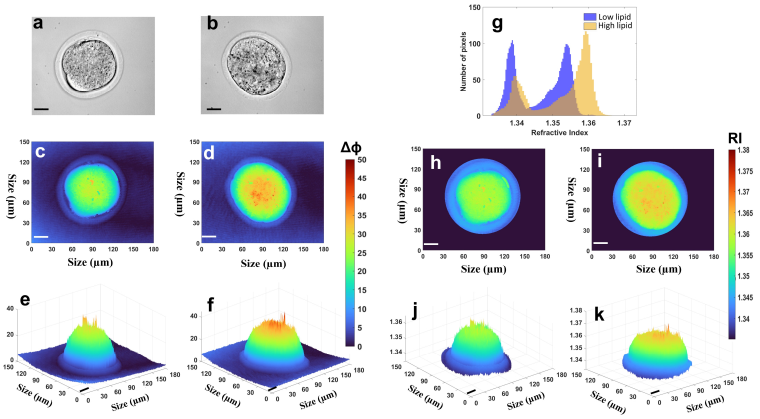

Fig. S3. Representative phase information and refractive index maps for morula-stage embryos cultured in low or high lipid media. (a,b) Bright field images for low lipid and high lipid-treated morula-stage embryos, respectively. (c,e and d,f) Reconstructed 2D phase profiles and their corresponding 3D topographical profiles for low lipid and high-lipid group respectively. (h,j and i,k) Reconstructed 2D refractive index profiles for low lipid and high lipid group with their corresponding topographical refractive index profiles. (g) Frequency distribution of all pixels measured using the reconstructed 2D refractive index profiles for low and high lipid groups of embryos in (h and i, respectively). Scale bars = 30 μm.

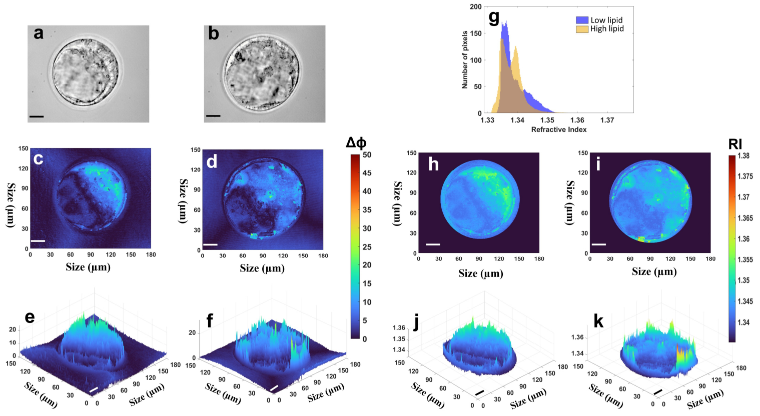

Fig. S4. Representative phase information and refractive index maps for blastocyst-stage embryos cultured in low or high lipid media. (a,b) Bright field images for low lipid and high lipid-treated blastocyst-stage embryos, respectively. (c,e and d,f) Reconstructed 2D phase profiles and their corresponding 3D topographical profiles for low lipid and high-lipid group respectively. (h,j and i,k) Reconstructed 2D refractive index profiles for low lipid and high lipid group with their corresponding topographical refractive index profiles. (g) Frequency distribution of all pixels measured using the reconstructed 2D refractive index profiles for low and high lipid groups of embryos in (h and i, respectively). Scale bars = 30 μm.
